# Early Morning Food Intake as a Risk Factor for Metabolic Dysregulation

**DOI:** 10.3390/nu12030756

**Published:** 2020-03-13

**Authors:** Ellen R. Stothard, Hannah K. Ritchie, Brian R. Birks, Robert H. Eckel, Janine Higgins, Edward L. Melanson, Kenneth P. Wright Jr., Andrew W. McHill

**Affiliations:** 1 Sleep and Chronobiology Laboratory, Department of Integrative Physiology, University of Colorado Boulder, 3100 Marine Street, Boulder, CO 80309, USA; Ellen.Stothard@Colorado.EDU (E.R.S.); Hannah.Ritchie@colorado.edu (H.K.R.); Brian.Birks@colorado.edu (B.R.B.); 2 Research and Development, Colorado Sleep Institute, 4895 Riverbend Rd A, Boulder, CO 80301, USA; 3 Division of Endocrinology, Metabolism, and Diabetes, University of Colorado Anschutz Medical Campus, 12801 East 17th Avenue, Aurora, CO 80045, USA; 4 Department of Pediatrics, Children’s Hospital Colorado Anschutz Medical Campus, 13123 East 16th Ave, Aurora, CO 80045, USA; Janine.Higgins@childrenscolorado.org; 5 Division of Geriatric Medicine, University of Colorado Anschutz Medical Campus, 12631 East 17th Avenue, Aurora, CO 80045, USA; 6 Geriatric Research, Education, and Clinical Center, VA Eastern Colorado Health Care System, 1700 N Wheeling St, Aurora, CO 80045, USA; 7 Oregon Institute of Occupational Health Sciences, Oregon Health and Science University, 3222 SW Research Drive, Portland, OR 97239 USA

**Keywords:** sleep, circadian, shiftwork, glucose tolerance

## Abstract

Increased risk of obesity and diabetes in shift workers may be related to food intake at adverse circadian times. Early morning shiftwork represents the largest proportion of shift workers in the United States, yet little is known about the impact of food intake in the early morning on metabolism. Eighteen participants (9 female) completed a counterbalanced 16 day design with two conditions separated by ~1 week: 8 h sleep opportunity at habitual time and simulated early morning shiftwork with 6.5 h sleep opportunity starting ~1 h earlier than habitual time. After wake time, resting energy expenditure (REE) was measured and blood was sampled for melatonin and fasting glucose and insulin. Following breakfast, post-prandial blood samples were collected every 40 min for 2 h and the thermic effect of food (TEF) was assessed for 3.25 h. Total sleep time was decreased by ~85 min (*p* < 0.0001), melatonin levels were higher (*p* < 0.0001) and post-prandial glucose levels were higher (*p* < 0.05) after one day of simulated early morning shiftwork compared with habitual wake time. REE was lower after simulated early morning shiftwork; however, TEF after breakfast was similar to habitual wake time. Insufficient sleep and caloric intake during a circadian phase of high melatonin levels may contribute to metabolic dysregulation in early morning shift workers.

## 1. Introduction

Metabolic diseases, including obesity and type 2 diabetes, continue to increase in prevalence [1]. Insufficient sleep has been identified as a risk factor contributing to weight gain and type 2 diabetes [2], potentially due, in part, to food intake occurring at night [3,4] when circadian-driven physiological processes are not prepared for food intake [5]. 

Shift workers are chronically exposed to altered behavioral schedules of sleep and wakefulness that result in insufficient sleep and increased food intake during the biological night, as defined by high endogenous melatonin levels. Circadian misalignment, defined as wake–sleep and feeding–fasting behaviors occurring at inappropriate circadian times, has been shown to impair glucose tolerance [6,7] and decrease total daily energy expenditure [8,9]. Additionally, recent studies suggest that the circadian timing of food intake may have a larger impact on body composition than the local clock time of food intake [4,10]. If sustained, the imbalance resulting from this behavioral pattern may lead to weight gain over time. 

The current research literature is comprised predominately of overnight shiftwork studies, yet individuals who begin work in the early morning hours [i.e., between 04:00 and 07:00 [11]] make up the largest population of shift workers in the United States [12]. As overweight body composition is prevalent among shift workers [13], and early morning shiftwork has been associated with higher levels of fasting insulin resistance [14], it is possible that food intake during the biological night may be a mechanism that contributes to negative metabolic outcomes in this population.

The purpose of this study was to examine the impact of food intake during a simulated early morning shiftwork protocol on sleep, endogenous melatonin levels, and metabolic outcomes. Specifically, it was hypothesized that simulated early morning shiftwork, including food intake during the biological night, would negatively impact metabolic outcomes as compared to waking and food intake at habitual timing.

## 2. Materials and Methods 

Twenty-two non-obese, healthy adults (12 females, aged 23.0 ± 3.5 year, BMI 23.5 ± 2.0 kg/m^2^) were enrolled into the research protocol. Participants reported being free from any current medical or psychiatric diagnosis, medications, drugs, and were non-smokers. Participants were healthy as assessed by physical, psychological, and sleep disorder screenings and physical exam, blood chemistries, 12 lead clinical electrocardiogram, and urine toxicology. Volunteers had not participated in shiftwork within six months or traveled across more than one time zone within three weeks prior to study. The investigations were carried out following the rules of the Declaration of Helsinki of 1975 (https://www.wma.net/what-we-do/medical-ethics/declaration-of-helsinki/), revised in 2013 and approved by the University of Colorado Boulder Institutional Review Board, (ID #14-0018, initial approval February 06, 2014). Participants gave written informed consent and were compensated for their participation.

### 2.1. Pre-In-Laboratory Procedures

For one week prior to each visit, participants maintained consistent, habitual, self-selected 8 h sleep schedules. Adherence was verified via concordance of sleep–wake logs, call-ins to a time-stamped voice recorder, and wrist actigraphy. Caffeine and alcohol use were proscribed one week prior to and throughout the study. Urine toxicology and breath alcohol testing verified that participants were drug and alcohol free upon laboratory admission. Females also completed a urine pregnancy test at the medical screening and upon laboratory admission. For three days prior to the laboratory protocol, participants were instructed to refrain from physical activity other than activities of daily living, and consumed an energy-balanced diet, prepared by Clinical and Translational Research Center Nutrition Core. The energy content of the diet was determined using resting energy expenditure (REE) at the medical screening visit and an activity factor that reflected the habitual low level of physical activity (1.5). Timing of sleep and study procedures, including food intake, were scheduled relative to each participant’s habitual sleep timing to maintain relative consistency with individual circadian timing of sleep. 

### 2.2. In-Laboratory Procedures

**Figure 1:**
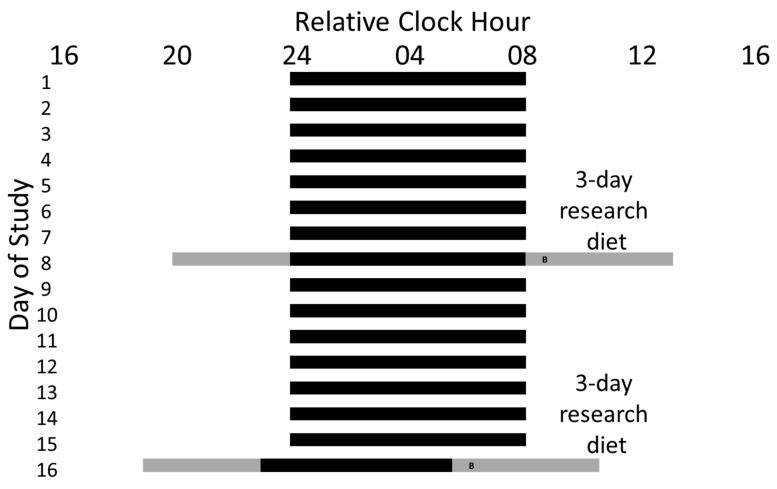
Example Study Protocol. A 16 day study protocol for a participant with a habitual sleep schedule of 24:00−08:00. The 8 h self-selected sleep schedules maintained at home are represented by black bars on days 1−7 and 9−15. During days 6−8 and 14−16, participants were provided an energy-balanced diet. Participants arrived at the laboratory on the evening of the 8th and 15th days of the study and were maintained in dim light for the study visit during scheduled wakefulness (represented by the gray bars) and darkness during scheduled sleep (represented by black bars). Participants were assigned first to either an 8 h sleep opportunity at their habitual time as a control condition or a 6.5 h sleep opportunity that began 1 h prior to habitual bedtime and ended 2.5 h prior to habitual wake time as a simulated early morning shiftwork condition. For each visit, participants completed baseline metabolic testing and blood samples were taken for metabolic and circadian markers after scheduled wake time. Participants were served an identical breakfast ~45 min after waking in each condition (represented by “B”) and post-meal testing continued for ~3 h.

Participants were tested under two counterbalanced study conditions in a crossover design (Figure 1): a habitual sleep condition and an early morning shiftwork condition, with half of the participants completing the early morning shiftwork condition first. Polysomnographic (PSG) recordings during sleep and wakefulness in both conditions were obtained using Siesta digital recorders (Compumedics). In the habitual sleep condition, participants were scheduled to an 8 h sleep opportunity at their habitual time. In the early morning shiftwork condition, participants were scheduled to a 6.5 h sleep opportunity from ~1 h prior to habitual bedtime until ~2.5 h prior to habitual wake time. These times were selected based on surveys and actigraphy-derived bed and wake times of early morning shift workers conducted by our laboratory (unpublished).

After scheduled wake time in both conditions, study procedures were identical with participants remaining seated in a semi-reclined (~35 °) position in dim light ( < 8 lux maximum at 183 cm in the direction of the ceiling fixtures and ~1.9 lux, ~0.6 Watts/m^2^ in the angle of gaze) for accurate assessment of melatonin levels under controlled conditions. Blood was drawn from an indwelling venous catheter placed after scheduled wake time for melatonin, fasted glucose, and insulin levels. Baseline REE was measured using standard indirect calorimetry with the ventilated hood technique (TrueOne® 2400, ParvoMedics, Sandy, UT). Respiratory gas exchange was measured for up to 30 min, depending on testing opportunity and values from the first stable 10−20 min were used to determine REE. After the baseline metabolic test, participants were served an identical (within-participant) breakfast 45 min after scheduled wake time in each condition which consisted of 25% of individual daily caloric needs. Participants were given 15 minutes to consume the meal. Blood was then sampled every 40 min for the next 2 h and metabolic testing was conducted every 45 min, beginning 15 min after breakfast, for 3.25 h to measure the thermic effect of food (TEF).

### 2.3. Analysis

Sleep staging criteria were defined according to standard guidelines of the American Academy of Sleep Medicine [15]. Sleep onset latency (SOL), was scored as time from lights out to the onset of three continuous epochs (SOL 1.5 min) or twenty continuous epochs (SOL 10 min) of PSG-defined sleep. Wakefulness after sleep onset (WASO) was defined as minutes of wakefulness after SOL 1.5 min. Latency to rapid-eye movement (REM) and slow-wave (SWS, stage 3/4) sleep were defined as time from SOL 1.5 min. Number and average duration of awakenings after SOL 1.5 min were calculated.

Blood samples were processed immediately, centrifuged and frozen at −70 °C until assayed. Melatonin and insulin were measured using radioimmunoassay (RIA melatonin; Rocky Mountain Diagnostics sensitivity 2.3 pg/mL; intra- and interassay coefficients of variation 11.0% and 10.7%, respectively, RIA insulin; sensitivity 3 uU/mL; intra- and interassay coefficients of variation 5.2% and 9.8%, respectively Colorado Springs, CO, Millpore, respectively) and glucose was assayed using hexokinase, UV (sensitivity 10 mg/dL; intra- and interassay coefficients of variation 0.67% and 1.44%, respectively, Beckman Coulter). 

Data from one participant were not included due to blood sampling difficulties and three participants completed only one visit. Thus, 18 participants (9 female) contributed to the final analysis. Mixed model ANOVA (STATISTICA V10, StatSoft) was used to examine changes in melatonin, insulin, glucose, and metabolic testing outcomes (REE, TEF) with condition and sample time as fixed factors and participant as a random factor. Sex and order were initially included in models to test impact on variables of interest and none were significant, thus were removed for final analyses. Insulin and glucose were also analyzed as the homeostatic model assessment of insulin resistance (HOMA-IR) and TEF data were as incremental area under the curve (iAUC) analysis. Planned comparisons using dependent t-tests were used to examine differences between conditions at individual time points. Data are presented as mean ± standard error of the mean (SEM). Effect sizes for condition and condition x time, generalized eta squared (*η*^2^_G_) were calculated using sum of squares from the mixed-effects ANOVA model accounting for variance due to individual differences by including subject as a random factor [16,17,18]. Standard benchmarks for small (0.02), medium (0.15) and large (0.35) effect sizes when using eta squared (*η*^2^) were used, even though effects for *η*^2^_G_ will be smaller than for η^2^.

## 3. Results

### 3.1. Participant Characteristics

Of the 18 participants who contributed to the final analysis, 9 participants were female (50%), the average age was 23.2 ± 0.9 years, BMI was 23.7 ± 0.6, and body fat percentage was 28.6 ± 0.2%. Upon awakening during both conditions, participants had an average REE of 1.3 ± 0.1 kcal/min, fasting glucose of 85.7 ± 1.2 mg/dL, and fasting insulin of 10.1 ± 0.5 IU/mL.

### 3.2. Sleep and Circadian Outcomes

**Figure 2:**
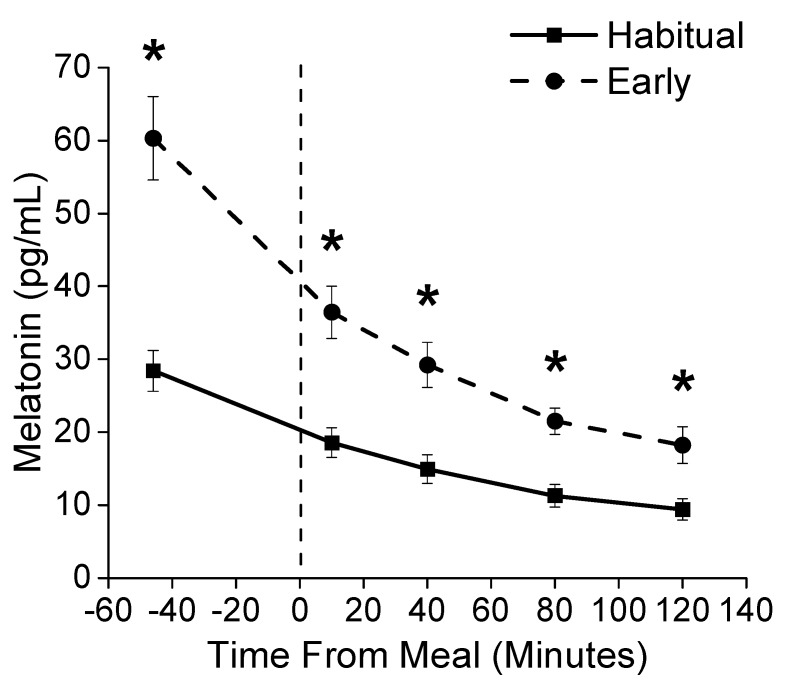
Melatonin Concentration. Plasma melatonin levels for the habitual sleep (squares) and simulated early morning shiftwork (circles) conditions. The vertical dashed line represents the start of the scheduled meal. * Denotes *p* < 0.05 between habitual sleep and simulated early morning shiftwork conditions.

Melatonin levels after waking were higher during early morning shiftwork compared to the habitual sleep condition (condition, *p* < 0.0001, medium effect size *η*^2^_G_ = 0.33; condition x time interaction, *p* < 0.0001, small effect size *η*^2^_G_ = 0.11; Figure 2). Planned comparisons showed that melatonin levels were higher at each time point measured in the early morning shiftwork condition (Figure 2).

Additionally, participants slept ~85 min less in the simulated early morning shiftwork compared to the habitual sleep condition (Table 1, large effect size *η*^2^_G_ = 0.79). This reduction in total sleep time was a result of decreased time spent in Non-Rapid Eye Movement (NREM) Stage 2 and Rapid Eye Movement (REM) sleep (Table 1, large effect sizes *η*^2^_G_ = 0.46 and *η*^2^_G_ =0.59, respectively). 

### 3.3. Metabolic Outcomes

**Figure 3:**
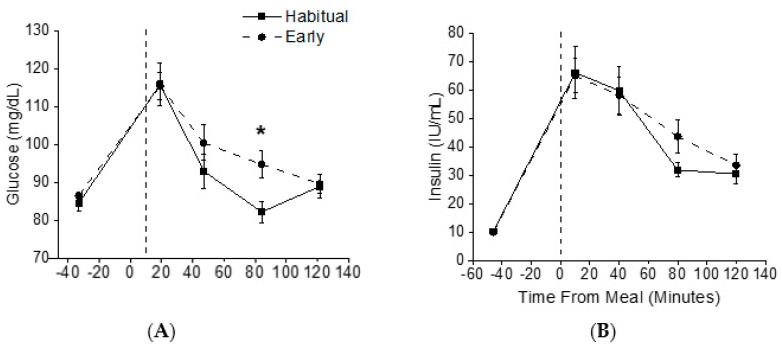
Glucose and Insulin Concentrations. Plasma glucose (**A**) and insulin (**B**) levels during habitual sleep (squares) and simulated early morning shift work (circles) conditions. The vertical dashed line represents the start of the scheduled meal. * Denotes *p* < 0.05 between time points.

Fasting insulin and glucose levels were similar between conditions. Post-prandial glucose levels were significantly elevated after food intake during the simulated early morning shiftwork compared to the habitual sleep condition (main effect of condition, *p* <0.05, small effect size *η*^2^_G_ = 0.03; Figure 3). Planned comparisons showed glucose levels were ~5% higher at 80 min after the meal in the early morning shiftwork condition (Figure 3). Insulin levels were similar between conditions (*p* = 0.31; Figure 3). There was no significant difference in HOMA-IR between conditions (*p* = 0.84).

**Figure 4:**
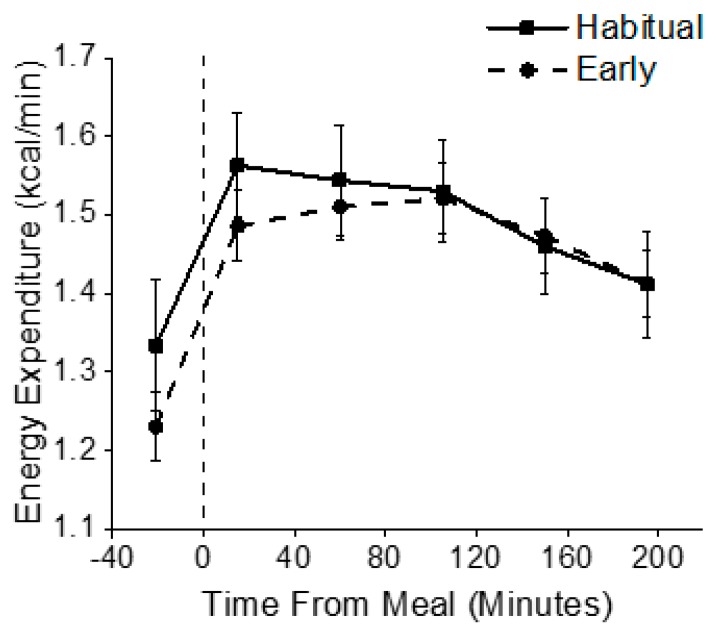
Energy Expenditure. Energy expenditure was assessed upon waking (REE fasted) and for 3.25 h after an identical test breakfast (TEF) in both habitual sleep (squares) and simulated early morning shiftwork (circles) conditions. The vertical dashed line represents the start of the scheduled meal.

Although there was a significant condition x time interaction (*p* < 0.01) for EE, the effect size was less than a small effect size (*η*^2^_G_ = 0.01) and none of the planned comparisons for REE or TEF between conditions at individual time points were significant before or after food intake, respectively (all *p* > 0.13; Figure 4), including iAUC analysis (*p* = 0.88).

## 4. Discussion

Obesity and diabetes are associated with negative health outcomes and are increasingly prevalent among the general population. Shift workers have an increased risk for developing these metabolic diseases, yet the mechanisms underlying this increased risk are unclear. Findings from the current study show that early morning caloric intake after one night of insufficient sleep, when melatonin levels are high, leads to a small increase in glucose levels when compared to the same meal after sleeping and awaking at habitual times. Additionally, EE was similar between conditions. 

The simulated early morning shiftwork protocol used in the current study was based on observational data from real-world early morning shift workers and, thus, allowed for examination of potential metabolic consequences associated with sleep–wake behaviors that commonly occur in this population. In the early morning shiftwork condition, participants slept ~85 min less and had higher melatonin levels for 2 h after wake time compared to the habitual sleep condition. This is consistent with other studies showing decreased sleep duration in early morning shiftwork [19], and was anticipated as the early morning shiftwork protocol curtailed the sleep opportunity by 90 min compared to habitual times. Moreover, by curtailing the majority of the sleep opportunity prior to habitual wake time, we primarily reduced participant’s sleep at a circadian phase in which sleep predominately alternates between Stage 2 and REM sleep [20]. This curtailment of early morning sleep may account for the observed differences in Stage 2 and REM sleep between groups and their association with melatonin concentrations. In regards to circadian timing, a melatonin threshold of 10 pg/ml in plasma is commonly used as a marker of the onset and offset of the biological night in humans [21]. Although melatonin levels were higher in the early morning shiftwork than habitual sleep condition, melatonin levels were, on average, still above the 10 pg/ml threshold in the habitual sleep condition. This finding is consistent with prior research showing that melatonin levels remain high for hours after habitual wake time in the modern environment [22,23]. As a result, the morning meal in the control condition also occurred during the biological night in the current protocol. 

Nightshift workers have been shown to have increased risk of cardiovascular and metabolic diseases [24,25,26,27], with in-laboratory studies highlighting circadian misalignment and sleep disruption as mechanisms for reduced glucose tolerance [6,9]. Notably, Morris and colleagues found a 6% increase in two-hour post-prandial glucose in the presence of a 14% elevation in late-phase post-prandial insulin when exposing participants to a simulated nightshift protocol that resulted in combined sleep restriction and circadian misalignment, indicating a possible reduction in insulin sensitivity [6]. The current study, which also induced combined circadian misalignment and sleep restriction, found that one night of early morning shiftwork in healthy adults increased post-prandial glucose levels without a subsequent increase in insulin. However, glucose levels in the healthy participants studied were still in the healthy range after breakfast. The pattern of increased post-prandial glucose with concurrent increased insulin has also been observed experimentally in chronic shift workers [28]. Findings of elevated post-prandial glucose during circadian misalignment may be of particular importance to the early morning shiftwork population due to their observed dietary choices. In a study of dietary profiles of different working schedules, Heath and colleagues found that workers starting shifts in the early morning consumed more carbohydrates daily compared to workers on other shifts [29]. Moreover, in qualitative interviews with nurses working nightshifts, shift workers have reported coping with feeling tired during the nightshift by consuming additional sugar for energy [30]. It is possible that chronic exposure to early morning schedules, as is common in hospital and other shiftwork schedules, combined with an increased consumption of high glycemic foods, could result in larger impairments in glucose metabolism over time than observed in this study. Additional research is needed to assess metabolic outcomes following chronic early morning shiftwork (i.e., recurring and chronic early morning shiftwork following multiple days of work and after recovery on free days) and should include more sensitive tests of glucose metabolism (i.e., intravenous glucose tolerance tests or hyperinsulinemic-euglycemic clamps).

In addition to glucose tolerance, the current study sought to determine the impact of one day of simulated early morning shiftwork on components of energy metabolism. Energy expenditure is an important component in weight maintenance, including 24 h energy expenditure, REE, and the TEF. In the current study, REE was lower after waking in the early morning shiftwork condition, then increased after breakfast to become similar to that of the habitual sleep condition. The finding of lower REE is similar to previous work performed by our laboratory. In that study, participants were studied in a simulated overnight shift work schedule in a whole-room indirect calorimeter. Findings demonstrated that total daily EE was decreased on the simulated nightshifts compared to a dayshift, primarily due to lower EE during daytime sleep following simulated night shifts [8]. REE is known to vary depending on circadian phase, with the lowest levels of EE occurring during the biological night [31,32,33]. Taken together, these findings suggest that EE is altered by the circadian timing of wakefulness and sleep episodes. If a lower EE were to persist chronically and if energy intake were to remain stable, it could contribute to a decrease in total daily EE, a state of positive energy balance, and weight gain, which has been reported in nightshift workers [34,35]. How chronic exposure to early morning shift schedules alters total daily EE and body composition is unknown.

Another component of total daily EE, the TEF, has previously been shown to vary based on time of day [6,36]. Specifically, we and others have also reported that the TEF is lowest in response to a meal consumed in the evening [8,36,37], which could contribute to the observation that those who eat during the biological night tend to have higher levels of body fat composition [4,10]. Our finding of no difference in TEF between conditions is inconsistent with our hypothesis that the TEF would be lower during simulated early morning shiftwork, when endogenous melatonin levels were elevated, as compared to habitual timing. Previous work by Romon and colleagues showed that the TEF is highest in the early morning hours [36], although they did not have any measure of circadian timing and studied all individuals at the same clock hour. One potential explanation for the similarities in the TEF between habitual and simulated early morning shiftwork conditions in the current study is that the two-hour advance in wake time may not have induced a sufficient circadian misalignment to detect meaningful differences in the TEF or that the endogenous melatonin levels in the control habitual sleep condition were still elevated, indicating the biological night. Future research is needed to identify mechanisms for the observed time of day differences in REE and TEF. 

We used mixed meal testing to examine the metabolic impacts of simulated early morning shiftwork with induced sleep and circadian misalignment on metabolic and energy expenditure outcomes. Findings from this initial study provide insight into the metabolic challenges that the largest population of shift workers face in real-world settings. However, it is important to consider the limitations and potential confounders of our protocol. First, young and healthy participants were studied and, thus, the generalizability of the findings to shift-working populations who, as mentioned previously, have additional disease burden is not represented in our population. Additionally, we cannot exclude a potential influence of sex differences on these outcomes given that our study was not designed with a sample size to test sex differences. Furthermore, we did not control for menstrual cycle across visits in the females tested. This may be of importance, as Qian and colleagues have recently demonstrated that there may be an impact of sex differences on metabolic outcomes in circadian misalignment [38]. Further investigation is needed with a sufficiently powered sample size to uncover potential sex differences during simulated early morning shiftwork. Finally, this protocol focused on the acute impact of only one night of sleep and circadian disruption on metabolic and energy metabolism outcomes, which does not allow this protocol to examine the impact of chronic exposure to such schedules, as is common in shift-working populations.

## 5. Conclusions

In summary, findings from this simulated early morning shiftwork protocol demonstrate possible implications for the metabolic health of early morning shift workers, particularly if these changes were to persist and worsen over time. Moreover, these findings indicate the need for future analysis exploring whether delaying breakfast time after an early morning awakening may be a potential therapeutic target for improving metabolic health in the largest population of shift workers, and perhaps in modern society for people with high morning melatonin levels for hours after awakening [22,23].

## Figures and Tables

**Table 1:** Sleep Architecture.

Parameter Minutes of Recording Time	Habitual Sleep	Simulated Early Morning Shiftwork	*p* Value
Stage 1	16.3 ± 1.7	15.2 ± 1.6	0.63
Stage 2	235.4 ± 5.0	182.3 ± 8.9	*p* < 0.0001
Stage 3/4 (SWS)	76.9 ± 6.9	84.8 ± 7.9	0.11
REM	107.3 ± 4.6	68.4 ± 3.6	*p* < 0.0001
Total Sleep Time (TST)	435.8 ± 5.8	350.7 ± 6.0	*p* < 0.0001
Sleep Efficiency (SE)	90.8 ± 1.2	88.7 ± 1.0	0.14
SOL 1.5 min	16.3 ± 3.3	16.4 ± 2.6	0.97
SOL 10 min	17.9 ± 3.3	19.2 ± 2.8	0.77
WASO from SOL 1.5 min	27.9 ± 4.1	28.3 ± 4.3	0.94
REML from SOL 1.5 min	109.7 ± 9.6	95.5 ± 9.0	0.33
SWSL from SOL 1.5 min	15.2 ± 1.5	14.0 ± 1.7	0.39
Number of Awakenings	21.4 ± 2.0	19.1 ± 1.9	0.24
Avg Duration of Awakenings	1.3 ± 0.2	1.5 ± 0.2	0.54

Data are presented as the mean ± SEM. Abbreviations are designated as follows: slow wave sleep (SWS); rapid eye movement (REM); sleep onset latency (SOL); wakefulness after sleep onset (WASO); latency to REM Sleep (REML); latency to SWS (SWSL).
